# Regulation of SARS-CoV-2 infection by diet-modulated gut microbiota

**DOI:** 10.3389/fcimb.2023.1167827

**Published:** 2023-06-29

**Authors:** Vivian Tieu, Sedra Tibi, Jun Ling

**Affiliations:** Department of Medical Education, California University of Science and Medicine, School of Medicine, Colton, CA, United States

**Keywords:** SARS-CoV-2, COVID-19, innate and adaptive immune system, microbiota, gut-lung axis, comorbidity, non-Western diet

## Abstract

Coronavirus disease 2019 (COVID-19) caused by SARS-CoV-2 infection has claimed millions of lives since late 2019, yet there are still many unexplored areas in its pathogenesis and clinical outcomes. COVID-19 is a disease that can affects multiple systems, some of which are overlapped with those modulated by gut microbiota, especially the immune system, thus leading to our concentration on analyzing the roles of microbiota in COVID-19 pathogenesis through the gut-lung axis. Dysbiosis of the commensal intestinal microbes and their metabolites (e.g., SCFAs) as well as the expression and activity of ACE2 in the gut could influence the host’s immune system in COVID-19 patients. Moreover, it has been known that the elderly and individuals diagnosed with comorbidities (e.g., hypertension, type 2 diabetes mellitus, cardiovascular disease, etc.) are more susceptible to gut flora alterations, SARS-CoV-2 infection, and death. Thus, in this review we will focus on analyzing how the gut microbiota regulates the immune system that leads to different responses to SARS-CoV-2 infection. Since diet is a major factor to modulate the status of gut microbiota, dietary influence on COVID-19 pathogenesis will be also discussed, aiming to shed light on how diet-modulated gut microbiota regulates the susceptibility, severity, and treatment of SARS-CoV-2 infection.

## Introduction

After nearly 3 years since the start of the SARS-CoV-2 global pandemic in 2019, there have been over 663 million cases and over 6.7 million COVID-19 related deaths worldwide (*WHO)*. Common clinical presentations of SARS-CoV-2 infection often involve the upper and lower respiratory tract, including fever, cough, dyspnea, sputum production, shortness of breath and respiratory failure. Although less commonly, patients may also have gastrointestinal symptoms ranging from diarrhea, nausea, vomiting, anorexia, ageusia, and hyposmia (Alimohamadi et al., 2020; CDC). In addition, patients infected with SARS-CoV-2 may present as asymptomatic or manifest with a variety of symptoms involving the renal, musculoskeletal, and cardiovascular systems (Alimohamadi et al., 2020; CDC). The large variation of COVID-19 symptoms is attributed to the high transmission and mutation rates as well as the unique targeting of ACE2 by SARS-CoV-2.

The immune system plays fundamental roles in protecting the host from foreign pathogens. However, the hyperactivation of the immune system can become problematic as seen in autoimmune disorders and other inflammatory states. Particularly, the pathogenesis of COVID-19 and the severity of its symptoms are highly associated with the “cytokine storm”. Cross talk between the microbiome of the gastrointestinal and respiratory systems referred to as the “gut-lung axis” also plays an important role in the SARS-CoV-2 pathogenesis (Batah and Fabro, 2021; Sencio et al., 2021a). The dysbiosis of the gut-lung axis may be critical to understand how acute respiratory distress syndrome (ARDS) develops in COVID-19 patients.

Comorbid diseases associated with increased risk of coronavirus infection include hypertension, chronic obstructive pulmonary disease (COPD), cardiovascular disease (CVD), hepatic diseases, diabetes mellitus (Types 1 and 2), malignancy, and obesity (Ejaz et al., 2020). Evidence also suggests that the elderly and minority ethnic groups constitute a majority of the COVID-19 patients and also have been diagnosed with the risk factors mentioned above (Kopel et al., 2020). Moreover, it has been recognized that “non-Western” diets, such as the Mediterranean diet, can shape the diversity and composition of the gut microbiome in patients with obesity, CVD, cognitive impairments (e.g., Alzheimer’s disease), and Type 2 Diabetes Mellitus (T2D) (Nagpal et al., 2019; Meslier et al., 2020; Breuninger et al., 2021; Rinott et al., 2022). This opens a possibility of utilizing nutritional and lifestyle interventions as preventative and treatment alternatives for COVID-19 considering the effects of comorbidities.

In this review, we focus on the connection between gut microbiota and the pathogenesis of SARS-CoV-2. Essential microbiome-derived metabolites such as short chain fatty acids (SCFAs) and ACE2 expression are key factors in regulating immune responses and the cytokine storm that leads to systemic inflammation (Zhang et al., 2022). Dysbiosis of the gut microbiome has been linked to the elderly population as well as in patients with T2D and CVD, comorbidities that have been tied to increased SARS-CoV-2 susceptibility and severity (Zhang et al., 2021). Thus, improvement of gut microbiome health through dietary interventions can serve as a preventative measure and an alternative treatment to minimize the severity of COVID-19 patients, especially for those who are considered “high-risk”.

## Dysregulation of innate and adaptive immune systems by SARS-CoV-2

SARS-CoV-2 can evade and/or downregulate the human innate immune system. The non-structural proteins (NSPs) of the coronavirus, such as NSP16 and NSP1, play a role in antagonizing PRR antigen recognition and the production of proinflammatory cytokines (Schultze and Aschenbrenner, 2021). Notably, there are decreased levels of the cytokines, Interferon Type I (IFN-α, IFN-β, IFN-ϵ, IFN-ω, and IFN-κ) and Interferon Type III (IFN-I) (Bastard et al., 2020). Additionally, patients with severe COVID-19 infections also produce neutralizing antibodies against Interferon Type I, specifically against IFN-α, IFN-ω, or both. Another study comparing the interferon levels of 155 COVID-19 patients found significantly elevated levels of IFN-Is and IFN-IIIs in the lower airways of patients (Sposito et al., 2021). The elevated IFN values have been associated with increased apoptosis and decreased cellular proliferation by activating pro-apoptotic p53 transcription factor, contributing to SARS-CoV-2 pathogenesis and cytokine storm. Interestingly, critically ill patients expressed markedly elevated IFN levels but reduced induction of protective IFN-stimulated genes (ISGs). In addition, there is a delayed induction and response of protective ISGs in elderly patients (≥70 years), thereby contributing to the increased risk of developing severe infection in this population.

The dysregulation of PRRs and IFNs prevent early detection of the SARS-CoV-2 virus by the immune system, allowing its unhindered replication and dissemination. However, it is important to recognize that a defect of the innate immune system is patient-specific and differs between mild/asymptomatic and life-threatening cases. It is also significant to consider that certain populations such as the elderly and the immunocompromised are especially vulnerable to SARS-CoV-2 infection with increased susceptibility and severity (Sette and Crotty, 2021). Furthermore, without a robust innate immune system, the host’s ability to prime its adaptive immune system is also diminished.

Several studies have shown how CD4+, CD8+, and regulatory T cells are significantly impaired in severe cases of infection (Paces et al., 2020). However, others have also demonstrated that in acute cases, CD4+ and CD8+ T cell levels are elevated and have been shown to be produced at a more rapid rate than in a healthy patient (Sette and Crotty, 2021). T-cell response is critical in clearing viral infection, which is correlated with an observation that more life-threatening cases are associated with decreased levels of SARS-CoV-2 CD4+ T cells. Depletion of CD4+ T cells in severe infections signifies the immune system’s inability to mediate antibody and CD8+ T cell responses, effector cell differentiation, and tissue repair. The trends observed with T cell levels and COVID-19 severity suggest that SARS-CoV-2 exhibits immunoevasive ability through disrupting normal antigen-presenting function via MHCs and downregulating T cell activity. Thus, the evasion of the immune system makes this novel coronavirus so deadly. Moreover, the dysregulation of both immune systems is responsible for the cytokine storm seen in severe cases.

## Cytokine storm

The cytokine storm is defined as an aggressive proinflammatory state of the host’s immune system and is associated with systemic complications. As a result of systemic inflammation, patients may present with overarching symptoms ranging from constitutional symptoms to vascular, renal, gastrointestinal, neuropsychiatric, cardiac, and respiratory symptoms. The severity and poor patient prognosis of COVID-19 is attributed to the cytokine storm. The increased levels of cytokines, chemokines, and inflammatory cells are responsible for the leading cause of death in COVID-19 patients: acute respiratory distress syndrome (ARDS). ARDS is characterized as a severe lung injury with alveolar damage, pulmonary edema, and progressive respiratory failure (Hashemian et al., 2021; Sadeghi et al., 2021). There have been increased efforts to prevent cytokine storm through antibody and stem cell treatment, aiming to decrease lung injury due to ARDS in patients (Galván-Román et al., 2021).

Since SARS CoV-2 can downregulate both innate and adaptive immune systems as described above, the imbalance of the host’s immune system is likely a mechanism to trigger the subsequent hyperinflammatory events in patients to “overcompensate” the initial downregulation of the immune system, thereby combating the continuously increasing viral load (Batah and Fabro, 2021). Although the cytokine storm can be considered as an attempt to restore immune function and balance, it is a significant contributor to COVID-19 severity and death. As a result, cytokine profiling is practically important to assist diagnosis and treatment, collectively improving the survival rates of COVID-19 patients.

An early study compared the cytokine levels in 41 COVID-19 patients. It was found that there were elevated plasma levels of IL1B, IL1RA, IL7, IL8, IL9, IL10, basic FGF, GCSF, GMCSF, IFNγ, IP10, MCP1, MIP1A, MIP1B, PDGF, TNFα, and VEGF in infected patients versus healthy controls (Huang et al., 2020). Moreover, the plasma levels of IL2, IL7, IL10, GSCF, IP10, MCP1, MIP1A, and TNFα were increased when comparing more severe (ICU) cases with non-ICU cases. Additional studies have also revealed that patients with severe COVID-19 have a significantly elevated cytokine profile of IL-2, IL-6, IL-7, IL-10, IP-10, MCP-1, TNF-α, macrophage inflammatory protein 1 alpha, and granulocyte-CSF compared to patients with mild to moderate COVID-19 (Hu et al., 2021). The marked increase in these cytokines is closely related with the inflammatory damage induced by SARS-CoV-2. Thus, they can serve as the targets for intervention before ARDS development.

A retrospective study examined 146 COVID-19 patients to determine if IL-6 is a sensitive and specific marker for disease severity (Galván-Román et al., 2021). The results demonstrated that patients with elevated IL-6 levels (>30 pg/mL) required mechanical ventilation and early Tocilizumab (anti-IL-6R antibody) treatment, suggesting that IL-6 is a suitable prognostic marker. It is also important to note that a majority of the patients included in this study were of an older demographic; most of them were diagnosed with other comorbidities such as cancer, CVD, hypertension, and diabetes mellitus. Another clinical study also showed increased levels of IL-6 and IL-10 amongst 32 COVID-19 patients who are now deceased due to SARS-CoV-2 infection compared to those who survived (Varchetta et al., 2021). IL-6, a pleiotropic cytokine, is broadly involved in the regulation of the immune system and is responsible for the activation of other cytokines and inflammatory processes. Elevated levels of IL-6 increase C-reactive protein synthesis and disrupt T-cell regulation and macrophage response, making it an important biomarker in determining the severity of COVID-19. Although there are elevated levels of anti-inflammatory cytokines such as IL-10, the outcompeting pro-inflammatory response during the cytokine storm curbs the immune system’s attempts back to homeostasis. The imbalanced cytokine profile in severe cases sheds light on the pathogenic role of pro-inflammatory cytokines in COVID-19 and the dangers when there are no effective regulatory mechanisms to keep the hyperinflammatory state in check. Thus, the unique cytokine profiling of healthy controls versus COVID-19 patients and severe COVID-19 cases versus mild to moderate cases provides insights into targeting the cytokine storm as a significant turning point in the treatment and management of COVID-19 patients.

## Gut-lung axis in COVID-19

The healthy gut microbiota population primarily consists of the *Firmicutes* (e.g., *Lactobacillus, Bacillus, and Clostridium*) and the *Bacteroidetes* species (e.g., *Bacteroides*) (Sencio et al., 2021a). The relationship between the gut microbiome and the host’s health status has been shown to be symbiotic. As a result, any disruption in this relationship may have diverse and profound effects as studied in cardiovascular disease, diabetes mellitus, malignancies, Alzheimer’s disease, cystic fibrosis, and upper respiratory infections such as COVID-19. Dysbiosis of the microbial population may provide insight into the pathogenesis of these disorders and offer alternative ways to treat them.

The gut-lung axis is the bidirectional crosstalk between the microbial community and the respiratory system. Any disruptions to the gut and/or lung will have consequences on both systems (Sencio et al., 2021a). Respiratory inflammation and damage may disrupt intestinal microbiota, and changes to the gut microbial composition may impact the function and mucosal immunity of the lungs (de Oliveira et al., 2021). It has been shown that the ratio between pro- and anti-inflammatory gut flora plays an important role in modulating immune homeostasis of the body (Wu et al., 2021).

Emerging studies now aim to investigate the specific mechanisms in which the gut microbiota contributes to establishing appropriate immune responses (immunomodulation) and homeostasis in the lungs. Gut-derived metabolic by-products and compounds such as Short Chain Fatty Acids (SCFAs) and the expression of Angiotensin Converting Enzyme 2 (ACE2) are considered key players in directing communication between gut microbiota and the lung immunity (Sencio et al., 2021a).

### Immunological effects of gut microbiota derived SCFAs

SCFAs are a group of fatty acid metabolites produced by gut microbiota during the anaerobic fermentation of indigestible polysaccharides. Acetate, butyrate, and propionate are the three main SCFAs absorbed by intestinal epithelial cells and distributed to the rest of the body (Valdes et al., 2018). SCFAs are involved in a variety of processes including hepatic glucose and lipid homeostasis, and appetite modulation. In the gut, SCFAs maintain intestinal barrier integrity by regulating inflammatory chemotaxis, differentiation, and proliferation as well as the production of cytokines, such as IL-8 which supports inflammation and facilitates epithelial integrity (Corrêa-Oliveira et al., 2016). In particular, therapeutic levels of SCFAs play a significant role in regulating inflammatory response, for which a healthy set of commensal microbiota is essential. Gut dysbiosis is associated with disrupted levels of SCFAs (Valdes et al., 2018), further affecting diverse immunological disorders or conditions ranging from autoimmune diseases to microbial infections (Erny et al., 2021; Liu et al., 2021; Yip et al., 2021).

Butyrate has been observed to stimulate regulatory T cell (Treg) production (Akimova et al., 2012), a key player in suppressing immune overactivation and maintaining self-tolerance (Scheinecker et al., 2020). Disruptions to Treg cell function or quantity have been associated with several autoimmune disorders (Miyara et al., 2011). A study by Arpaia et al. (2013) found that mice with a normal set of commensal bacteria had increased levels of butyrate and propionate, leading to the increased extrathymic differentiation of Treg cells compared to antibiotic-treated and germ-free mice. Treatment with butyrate in drinking water resulted in an increase in peripheral Treg cells in antibiotic-treated mice. Further studies found consistent associations between SCFAs and Treg cell homeostasis (Coutzac et al., 2020; Su et al., 2020).

SCFAs are also believed to promote the expression of IFN-β, a cytokine that enhances the expression of anti-inflammatory IL-10 and transcription of antiviral genes (e.g., MHC II, TLR-induced cytokines), to decrease levels of proinflammatory markers (e.g., IL-6, IL-12) in the lung, and to provide defense against Respiratory Syncytial Virus (RSV) infection (Karimi et al., 2020). Antunes et al. (2022) observed that treatment of human pulmonary epithelial cells with acetate showed protection against RSV. This finding is supported by another study which observed probiotic mice having increased levels of acetate in circulation and increased production of IFN-β by alveolar macrophages compared to non-treated mice (Ji et al., 2021). These studies demonstrate that the gut derived SCFAs can exert an anti-inflammatory effect to prevent the overreaction of immune responses.

### ACE2 expression

The Angiotensin Converting Enzyme 2 (ACE2) is a protein located on the plasma membrane of many cells in the kidneys, heart, testes (Tipnis et al., 2000), lungs (Kuba et al., 2005), and small intestine (Hashimoto et al., 2012). In the cardiovascular and kidney systems, ACE2 plays an important role in regulating blood pressure by cleaving and deactivating Angiotensin II as part of the Renin-Angiotensin-Aldosterone System (RAAS) (Perlot and Penninger, 2013). ACE2 expression in the gastrointestinal tract is believed to play an important role in regulating gut microbiota and enhancing innate immunity. Studies have demonstrated the link between ACE2 and several immunological disorders including systemic sclerosis (Miziolek et al., 2021) and Diabetes Mellitus Type 1 (Roca-Ho et al., 2020).

The functions of ACE2 in the immune system and in the regulation of gut-lung axis have been extensively studied. Intestinal ACE2 is involved in maintaining gut microbial homeostasis via regulation of dietary amino acid digestion (Camargo et al., 2020) and amino acid transporters (Penninger et al., 2021). Specifically, ACE2 facilitates the uptake of dietary amino acids such as tryptophan, glutamine, and phenylalanine; these amino acids are further processed by gut microbiota. These ACE2 metabolites play crucial roles in attenuating excessive immune responses by downregulating pro-inflammatory cytokines, promoting tight junction formation between gut epithelial cells, and modulating mucosal cell autophagy (Obukhov et al., 2020). In a study conducted on mice, plasma levels of glycine, tryptophan, and other neutral amino acids were decreased in ace2 null mice compared to wildtype mice (Singer et al., 2012). A study by Hashimoto et al. (2012) demonstrated that dietary tryptophan absorbed in an ACE2-dependent pathway uses mTOR signaling to regulate the expression of antimicrobial peptides in the intestines, and ACE2 deficiency was associated with higher risk for colitis. In the same study, findings also revealed that ACE2-mutant mice had a remarkably altered gut microbiome, markedly reduced serum levels of tryptophan, and a correlated susceptibility to intestinal inflammation compared to the wildtype mice. A tryptophan diet for ACE2-deficient mice reverted the composition of the intestinal microbiome closer to that of the wildtype mice with observed inductions of intestinal antimicrobial peptides. Intestinal antimicrobial peptides play a role in maintaining microbial homeostasis in the intestines (Salzman et al., 2010). These findings suggest the critical functions of ACE2 metabolites in regulating gut microbial homeostasis and modulating the local and systemic immune systems.

### Effects of SCFAs and ACE2 on COVID-19

As stated earlier, cytokine storm largely contributes to SARS-CoV-2 related ARDS, severity, treatment, and outcome of COVID-19. As a part of the gut-lung axis, SCFAs and ACE2 play a key role in modulating the immune response during severe respiratory disease and reducing the risk for cytokine storm and ARDS. In several studies, COVID-19 patients with digestive symptoms were more likely to develop severe illness and ARDS that required mechanical ventilation compared to patients without digestive symptoms (Guan et al., 2020; Jin et al., 2020). This suggests the significance of gastrointestinal components in COVID-19 progression.

Enhanced plasma levels of SCFAs have been correlated to improved lung protection and immune response under respiratory infections by influenza (Sencio et al., 2021b), *Streptococcus pneumoniae* (Machado et al., 2022), and respiratory syncytial virus (Antunes et al., 2022). SCFAs have also been observed to minimize the repercussions of severe COVID-19 infection by potentially mitigating the cytokine storm (Reinold et al., 2021; Vignesh et al., 2021). Alterations in gut microbiome have been associated with COVID-19 infection, notably a decrease in butyrate-producing bacteria such as *Ruminococcaceae* and *Lachnospiraceae* families; these changes persist after the recovery of COVID-19 (Gu et al., 2020; Zuo et al., 2020). A study by Zhang et al. (2022) found that decreased abundance of SCFA-producing bacteria, such as *Bifidobacterium* and *Faecalibacterium*, was observed in more severe COVID-19 cases with deficient levels of SCFAs during infection that continued beyond 30 days after resolution. Findings by Li J et al. (2021) suggested that butyrate regulates gene expression through the deacetylation by histone-deacetylase (HDAC). These genes include the downregulation of high-mobility group protein-1 (HMGB1) that is essential for SARS-CoV-2 infection and replication (Bruchez et al., 2020) and the upregulation of antiviral gene pathways via toll-like receptor signaling and MHC class-II transactivator (CIITA), the latter of which is known for disrupting endosomal entry of the virus into gut epithelial cells (Bruchez et al., 2020). Butyrate's effect on histone arginine-demethylase *Jmjd6* and chromatin-remodeling complex members *Smarca4* and *Arid1a* reduces SARS-CoV-2 induced cell death, suggesting a protective role for gut epithelial cells. Further studies had similar findings in which depletions of SCFA-producing bacteria including *Faecalibacterium* (Zuo et al., 2020), *Roseburia* and *Bifidobacterium* (Yeoh et al., 2021) were associated with increased pro-inflammatory cell markers, lower levels of CD8+ T cells, and more severe COVID-19 infection (Reinold et al., 2021; Yeoh et al., 2021). Notably, reductions in SCFA-producing bacteria were found to be significantly correlated to increased pro-inflammatory cytokines, blood markers, and severity in COVID-19 patients (Yeoh et al., 2021).

**Figure 1:**
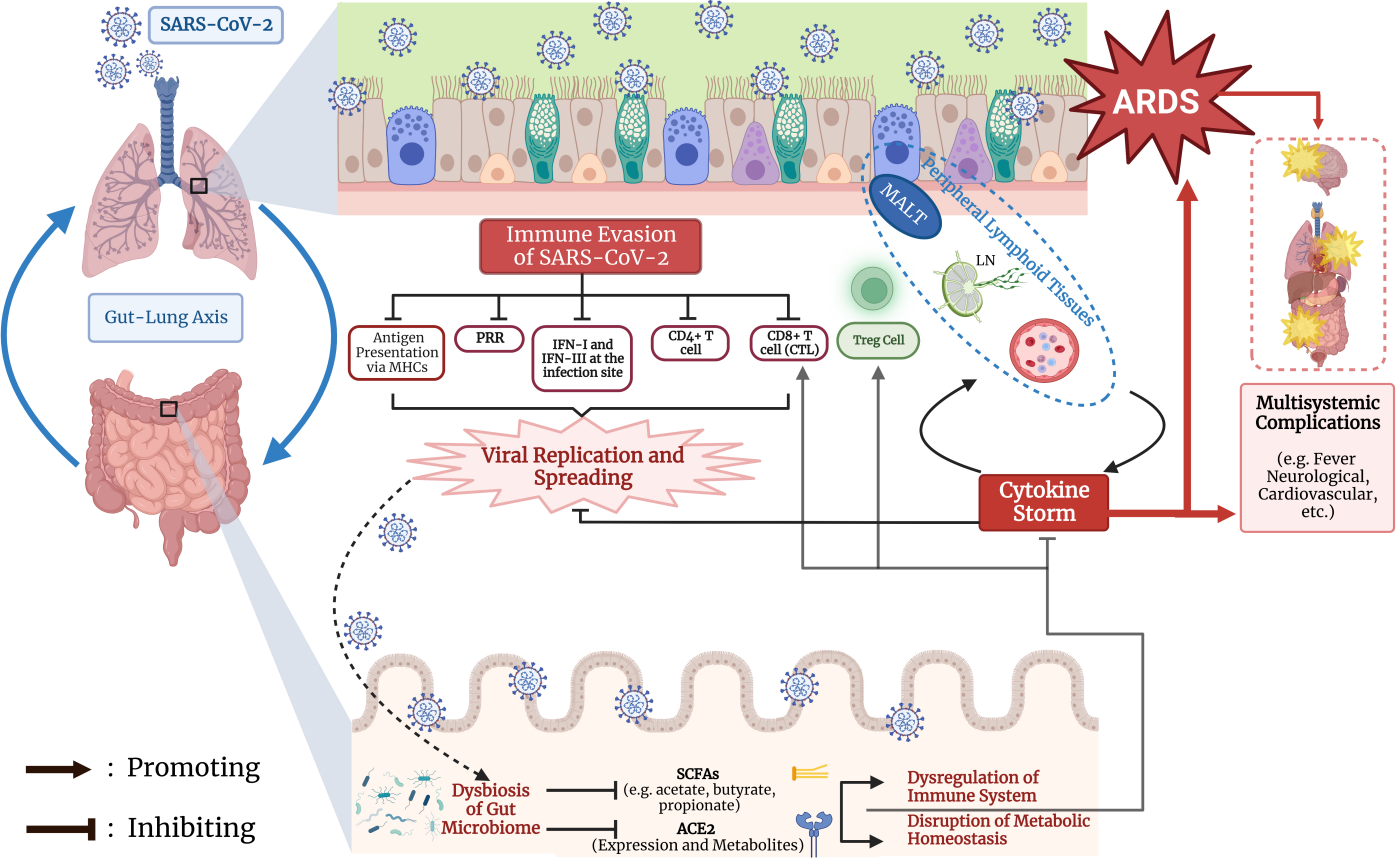
Regulation of COVID-19 progression and outcome by immunological interaction through gut-lung axis. The early event of immune evasion of SARS-CoV-2, the generation of dysbiosis of the gut microbiota, and synergistic effects between lung and gut on cytokine storm and T cell mediated immunity are summarized. MHC, major histocompatibility complex; PRR: pattern recognition receptor; IFN: interferon; CTL: cytotoxic T lymphocyte; Treg, regulatory T cell; MALT, mucosa-associated lymphoid tissue; LN, lymph node; ARDS, acute respiratory distress syndrome; SCFA, short chain fatty acid; ACE2, angiotensin converting enzyme 2. The Figure was created using BioRender software (https://www.biorender.com/).

The effect of COVID-19 on ACE2 expression in the gut may contribute to disease progression and outcome. After SARS-CoV-2 binds to the ACE2 receptor, it causes ACE2 degradation through viral internalization and replication (Hoffmann et al., 2020; Penninger et al., 2021), hindering its normal function in the uptake of dietary amino acids, most notably tryptophan (Qin et al., 2021). Reduced ACE2 expression decreases the levels of its free amino acid metabolites, leading to disruptions in the gut barrier and causing a “leaky gut” which allows bacterial lipopolysaccharides and peptidoglycan to enter systemic circulation. This further provokes the immune system during infection and contributes to the cytokine storm observed in severe COVID-19 patients (Penninger et al., 2021). When the gut-lung axis is disrupted by the downregulation of intestinal ACE2 under COVID-19, cytokine storm is more likely to arise that leads to ARDS and severe symptoms in the lungs. In summary, the gut microbiota derived SCFAs and the state of intestinal ACE2 are the two relatively well studied regulators in the gut-lung axis to modulate the immune responses against COVID-19. The whole immune response process and mechanisms for gut microbiota to regulate COVID-19 outcomes are summarized in **Figure 1**.

## Underlying comorbidities and gut dysbiosis as a part of COVID-19 pathogenesis

A study has found that COVID-19 morbidity and mortality are correlated with older age, diabetes mellitus type 2 (T2DM), and hypertension (Zhang, J et al., 2021). Interestingly, these same populations are predisposed to having alterations in gut microbial composition.

Gut microbial changes associated with the natural aging process could play a role in the development of immunosenescence. A study by Hopkins, et al. (2001) observed consistent patterns in the shift of gut microbiota composition in the elderly age group (aged 67-88 years), notably a decrease in SCFA-producing bacteria (*Lactobacillus, Bifidobacteria*) compared to the young adult group (aged 21-34 years). Woodmansey et al. (2004) found a significant decrease of acetate and butyrate in healthy elderly compared to antibiotic-treated elderly patients. This supports the use of probiotics in the elderly to enhance natural killer cell activity and quantity (Gui et al., 2020) and decrease IL-6, IL-8, and C-reactive protein (Costabile et al., 2017). A randomized trial found that COVID-19 patients who took probiotics had shorter duration of symptoms, lower viral loads, and a higher rate of complete remission compared to COVID-19 patients in the placebo group (Gutiérrez-Castrellón et al., 2022 *)*.

Individuals with T2DM have a higher risk for morbidity and mortality under many respiratory infections such as influenza (Lau et al., 2014), community-acquired pneumonia (Di Yacovo et al., 2013), and COVID-19. A metagenomic analysis found that gut dysbiosis in T2DM exhibited decreased levels of butyrate-producing bacteria, increased opportunistic strains, and increased oxidative stress (Qin et al., 2012). Probiotic supplementation in diabetic patients was found to restore acetate and propionate levels and moderately improve levels of SCFA-producing bacteria *Bifidobacteria* (Birkeland et al., 2020). Another study by Tonucci et al. (2017) found that replenished acetate levels in diabetic patients under probiotic supplementation were correlated reduction of pro-inflammatory and oxidative stress factors such as TNF-α, LDL-cholesterol, and HbA1c. When encountering an infection such as COVID-19, reducing immune provocation is critical to lower a risk for developing cytokine storm.

Patients with uncontrolled hypertension are more susceptible to developing severe COVID-19. Gao et al. (2020) observed that angiotensin-converting enzyme inhibitors (ACEi) and angiotensin II receptor blockers (ARBs) help reduce mortality in COVID-19 patients compared to those without antihypertensive treatment or with the use of non-RAAS inhibitors (beta blockers, diuretics, calcium channel blockers). ACEi and ARBs are believed to increase ACE2 expression in cardiac tissue (Ferrario et al., 2005) and intestinal enterocytes (Vuille-dit-Bille et al., 2015), which leads to the reduction of Angiotensin II levels. Ang-II mediated fibrosis and lung injury is believed to play a crucial role in worsening lung injury in COVID-19 (Kuba et al., 2005), therefore preservation of ACE2 using RAAS inhibitors may offer protection against SARS-CoV-2 injury (South et al., 2020). On the other hand, increased ACE2 expression may provide more host receptors for binding with SARS-CoV-2, leading to concerns of harmful ramifications of ACEi and ARBs (Fang et al., 2020). A study by Bauer et al. (2021) found that discontinuation of ACEi/ARBs led to a faster recovery in elderly COVID-19 patients in Germany. Li, S et al. (2021) found that the inpatient use of ARBs was associated with significant decreased mortality in COVID-19 African American patients. Other studies found no increased risk of mortality or morbidity with the use of ACEi or ARBs in hypertensive patients with COVID-19 (Gao et al., 2020). These findings may be controversial in some cases, suggesting that the effects of RAAS inhibitors on COVID-19 patients with hypertension may be protective or harmful depending on the intricate regulation between RAAS pathway and SARS-CoV-2 entry through ACE2. This issue is further complicated by the fact that ACEi can activate the kallikrein-bradykinin pathway; increased bradykinin level can induce hypotension, pulmonary edema, dizziness, myalgia, arrhythmia, etc., which could potentially cause the deterioration of COVID-19 patients (Wang et al., 2020). Collectively, ACEi and ARBs show more benefits than harms for COVID-19 patients with hypertension. Discontinuation of RAAS inhibitors should be cautiously determined based on specific patient situation.

## Diet modulates COVID-19 through gut microbiota

The popularity of the Western diets and processed foods worldwide have raised concerns as they are correlated with dyslipidemia, insulin resistance, overactivated renin-angiotensin system, overactivated sympathetic nervous system, and hyperinflammatory states (Beam et al., 2021). In several human and animal studies, the nutritional components of the Western diets (e.g., saturated fatty acids, cholesterol, and sugars) result in an increased oxidative state and increased levels of proinflammatory markers such as C-reactive protein (CRP), IL-1, and IL-6 (Christ et al., 2019). Interestingly, choline enriched in meat can be metabolized to trimethylamine (TMA) by gut microbes and further converted into trimethylamine-N-oxide (TMAO) in the liver. TMAO is now known to be detrimental to human health to cause many cardiovascular and metabolic diseases such as atherosclerosis, hypertension, diabetes, and heart failure, most of which are underlying comorbidities for COVID-19 (Shanmugham et al., 2023). Specifically, TMAO can induce IL-6 production and enhance the infection of endothelial cells by SARS-CoV-2 (Chiang et al., 2022). Thus, adopting “non-Western” diets, such as Mediterranean, high fiber, and fermented food diets, can alter the microbiome and improve metabolic and inflammatory processes in the gut.

Mediterranean diets have been shown to increase abundance of butyrate producers (e.g., *Agathobaculum butyriciproducens, Anaerostipes hadrus, Faecalibacterium prausnitzii*) and bacteria with anti-inflammatory properties (e.g., *Faecalibacterium prausnitzii, Roseburia*, and *Lachnospiraceae*) (van Soest et al., 2020; Barber et al., 2021). Similarly, high fiber diets provide some indigestible carbohydrates to produce SCFAs to maintain homeostasis of many physiological processes of the body (Hills et al., 2019; Wilson et al., 2020; Tanes et al., 2021; Wastyk et al., 2021). Adherence to a whole grain diet for 6 weeks resulted in an increase in effector memory T cells and the production of TNF-α amongst the 81 participants (Vanegas et al., 2017), suggesting that plant-based diets or the core component of blue-zones diets with non-processed plant-based foods could have protective role against SARS-CoV-2 infection. Indeed, this is also supported by recent studies directly examining the effects of plant-based diets on COVID-19 that show benefits to protect moderate-to-severe COVID-19 and to reduce the burden of long-COVID (Kim et al., 2021; Storz, 2021). Furthermore, patients subjected to synthetic enteral nutrition (EEN) demonstrated the importance of fiber for the recovery and maintenance of a healthy gut microbiome (Tanes et al., 2021). Fermented foods have been shown to modulate the gut microbiota composition and decrease inflammatory markers (e.g., C-reactive protein) and erythrocyte sedimentation rate (Bellikci-Koyu et al., 2019; Wastyk et al., 2021; Zhang, X et al., 2021). The anti-inflammatory and “protective” effects of the “non-Western” diets as discussed above may provide a means to treat hyperinflammatory diseases such as COVID-19 with cytokine storm.

A study conducted on a COVID-19 patient with a history of Graves’ disease, hypertension, hyperlipidemia, and prediabetes demonstrated that a high fiber diet alleviates GI symptoms post-acute infection (Wang et al., 2022). Moreover, there was increased abundance of SCFA producing bacteria, such as *Oscillibacter, Sellimonas, Bifidobacterium, Blautia, Lactobacillus, Faecalitalea, Anaerofustis*, and *Eubacterium*, after 2 months of the high-fiber dietary intervention.

Another study examined the efficacy of a probiotic formula on altering the gut microbiota and improving symptoms in 293 symptomatic COVID-19 outpatients (Gutiérrez-Castrellón et al., 2022). The results showed that 53.1% of patients in the probiotic group achieved complete remission from SARS-CoV-2 infection after 30 days, whereas only 28.1% of patients in the placebo group did. In addition, the viral load and symptoms were both reduced for patients in the probiotic group. Longitudinal studies should be conducted to assess the long-term efficacy of such dietary interventions.

## Discussion

Gut microbiome plays increasingly important roles in human pathophysiology through its interaction with other body systems, thereby emerging as a critical factor in regulating disease development, prevention, treatment, and outcome. In this review, we specifically analyze the function of gut microbiota in COVID-19 by focusing on how its metabolites like SCFAs and the status of intestinal ACE2 regulate the innate and adaptive immunities in the context of gut-lung axis. Aging, comorbidities, and diets as the major modulators of gut microbiota are also integrated for us to further understand the complex regulation between gut microbiota and COVID-19.

During the interaction between gut microbiota and immune responses, the function of intestinal ACE2 is an intriguing topic. Relative enrichment of ACE2 in the gut makes the GI tract a major organ susceptible to SARS-CoV-2 infection; on the other hand, maintenance of intestinal ACE2 expression is required for the healthy functions of the gut. Thus, the balance between these two counteracting roles of ACE2 become critical in regulating SARS-CoV-2 infection. One aspect of this balance is analyzed through the therapeutic effects of ACEi and ARBs on patients with comorbidities. Further studies are needed on this topic in the wake of COVID-19 pandemic.

The other critical point we emphasized in this paper is how gut eubiosis is compromised or disrupted by comorbidities and diets that lead to increased susceptibility to SARS-CoV-2 infections. Interestingly, SARS-CoV-2 infection can also cause gut dysbiosis, thereby synergizing the damaging cycle to favor its infection. Considering this topic, currently fecal microbiota transplantation (FMT) becomes a novel approach that is proved to be effective by transferring gut commensal microbes from a healthy person to a patient to improve gut microbial composition and homeostasis in the recipient (Wang et al., 2019). FMT has demonstrated promising effects in the use of *Clostridium difficile* infections (van Nood et al., 2013) to induce remission in active ulcerative colitis patients (Moayyedi et al., 2015), to control symptoms in irritable bowel syndrome patients (Johnsen et al., 2018), and to improve hepatic encephalopathy in cirrhotic patients (Bajaj et al., 2017). Zhang, W et al. (2020) observed that mice with colitis had increased levels of SCFAs, decreased levels of pro-inflammatory cytokines, and overall reduced inflammatory markers from FMT treatment. Considering these positive effects, FMT may become promising in treating COVID-19 in patients with gut dysbiosis caused by various comorbidities.

## Author contributions

VT, ST, and JL conceived the idea for this paper. VT and ST wrote the manuscript. JL supervised the research and revised the manuscript. All authors contributed to the article and approved the submitted version.
